# A Rare Presentation of Granulomatosis With Polyangiitis with Multiple Cardiac Valvular Insufficiencies

**DOI:** 10.7759/cureus.28617

**Published:** 2022-08-31

**Authors:** Ashley Aya, Ankita Prasad, Mina Aknouk, Smriti Kochhar, Arthur Okere

**Affiliations:** 1 Internal Medicine, Hackensack Meridian Health, Ocean University Medical Center, Brick, USA; 2 Medical School, Hackensack Meridian Health, Ocean University Medical Center, Brick, USA; 3 Medical School, Rowan School of Osteopathic Medicine, Stratford, USA; 4 Cardiology, Hackensack Meridian Health, Ocean University Medical Center, Brick, USA

**Keywords:** regurgitation, tricuspid regurgitation, mitral regurgitation, acute kidney injury, valvulitis, eosinophils, valvular deformity, necrotitisng vasulitis, cardiac, granulomatosis polyangitis

## Abstract

Granulomatosis with polyangiitis (GPA), earlier known as Wegener's granulomatosis, is an autoimmune inflammatory disorder that causes necrotizing vasculitis of small- and medium-sized blood vessels. It primarily affects the upper respiratory tract, lungs, and kidneys. Most of the cardiac involvement tends to be subclinical and is often not clinically apparent with involvement of the conduction pathway; myocarditis, pericarditis, or coronary artery involvement are associated with increased morbidity and mortality. These present with the symptoms of shortness of breath, cough, bilateral pedal edema, orthopnea, syncope, and features of heart failure such as elevated jugular venous pressure. We report a rare case of heart involvement with profound valvular deformity involving all four cardiac valves along with renal impairment in a 76-year-old female with recently diagnosed granulomatosis with polyangiitis.

## Introduction

Granulomatosis with polyangiitis (GPA) is an autoimmune disorder affecting small-and medium-sized blood vessels. The prevalence of GPA is 15.7 cases per 100,000 patients [1]. It primarily affects the upper respiratory tract, lungs, and kidneys. A higher percentage of cardiac lesions are found in patients with severe renal involvement. Most cardiac lesions are subclinical and involve the conduction pathway, myocarditis, pericarditis, or coronary artery involvement. Cardiac manifestations are often not clinically apparent but are associated with increased morbidity and mortality. Most patients with cardiac involvement remain clinically silent. Cardiomyopathy with reduced ejection fraction has been reported occasionally [2]. In 30% of patients with GPA, heart involvement has been reported at autopsy [3]. We report a rare case of cardiac involvement with profound valvular deformity in a 76-year-old female with granulomatosis with polyangiitis.

## Case presentation

A 76-year-old female presented to the emergency department (ED) after a fall at home. She denied head trauma, loss of consciousness, lightheadedness, dizziness, chest pain, and palpitations but admitted to some weakness over the past 6-8 weeks, which had worsened in the last 1-2 weeks. This, along with the recent fall, prompted her to be evaluated. She also had anorexia and unspecified weight loss in the last few weeks. Her past medical and surgical history was notable for breast cancer with mastectomy and hypothyroidism. She did not complain of fever, shortness of breath, palpitations, chest pain, bleeding, or noticeable swelling. She did not complain of oral or nasal ulcers, rashes, muscle weakness, paresthesias, belly pain, nausea, vomiting, or blood in her urine or stool. She was conscious, comfortable, alert, and mildly anxious at the presentation. She had no obvious signs of injury. Her vitals at admission were her blood pressure at 162/78 mmHg, pulse of 95 beats per minute, respiratory rate of 18 breaths per minute, oxygen saturation of 99% on room air, and temperature of 97.9 degrees Fahrenheit. On physical examination, heart sounds were normal with a regular rate and rhythm with no appreciable murmurs. Lung sounds were clear with no added sounds; the abdomen was soft with no organomegaly; bowel sounds were normally present, and there was no tenderness to palpation. There was no edema in the bilateral lower extremities. Laboratory investigations revealed leucocytosis with a count of 22.2 k/uL (4.5-11 mg/dL), hemoglobin 8.3 g/dL (12-16 g/dL), creatinine of 2.19 mg/dL (0.44-1.00 mg/dL), blood urea nitrogen (BUN) 33 mg/dL (5-25 mg/dL), sodium of 133 mmol/L (136-145 mmol/L), and serum albumin of 1.9 g/dL (3.5-5.0 g/dL). Urinalysis showed 3+ protein, moderate blood with 15-20 RBCs, and 8-10 WBCs.

**Figure 1:**
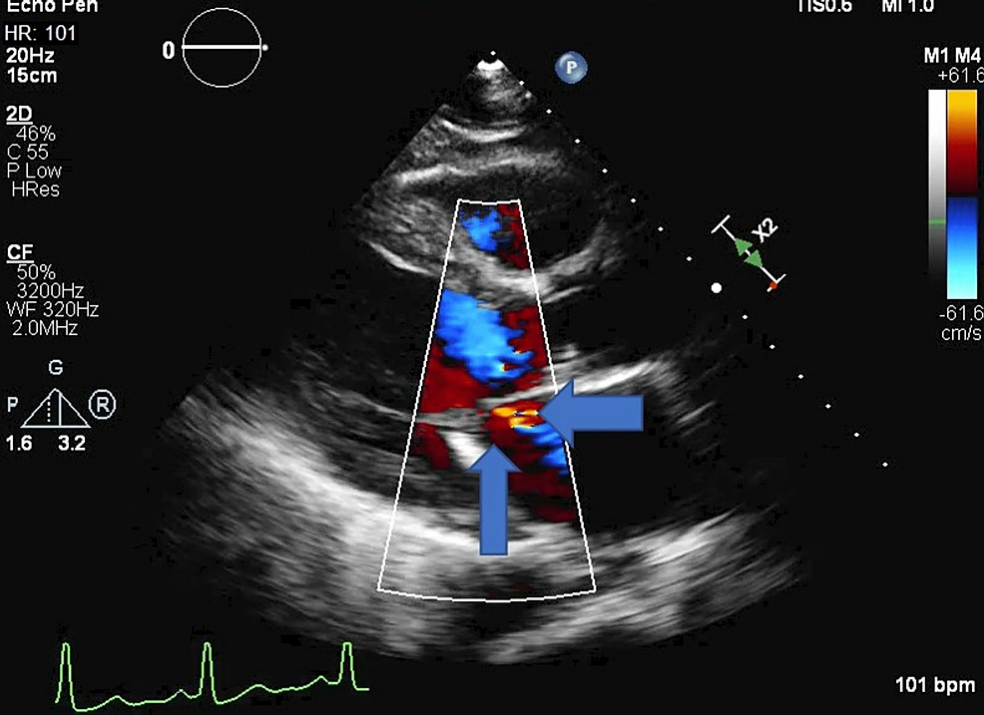
2D echo showing left atrial, left ventricle and right ventricle with blue arrows showing significant mitral regurgitation

**Figure 2:**
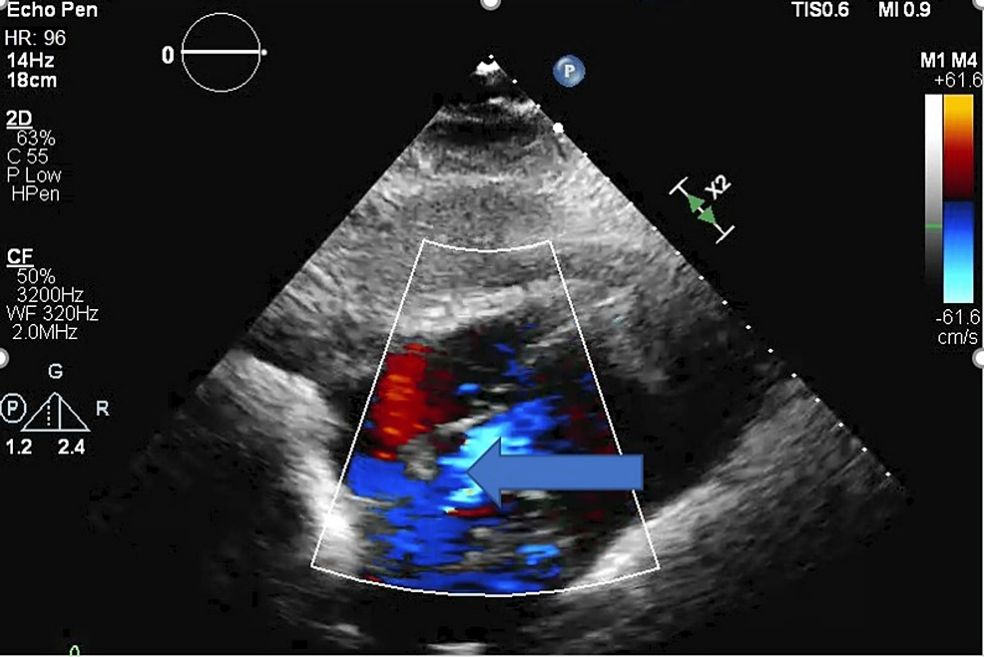
2D echo showing tricuspid regurgitation (blue arrow)

**Figure 3:**
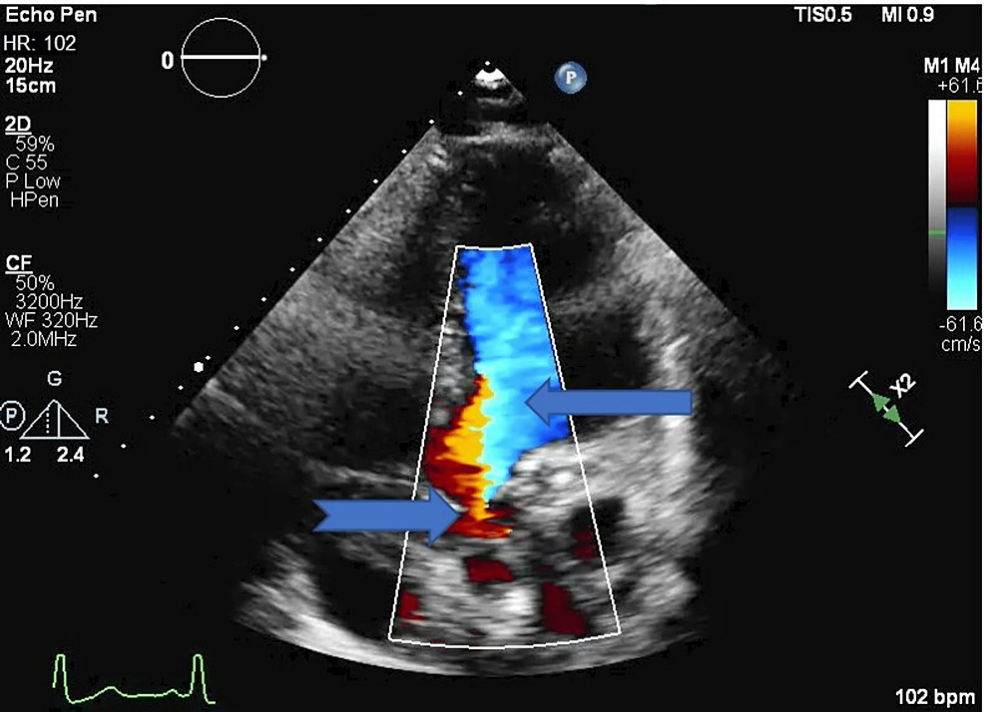
2D echo, five-chamber view showing aortic regurgitation (blue arrow with broken tail) and mitral regurgitation (blue arrow)

**Figure 4:**
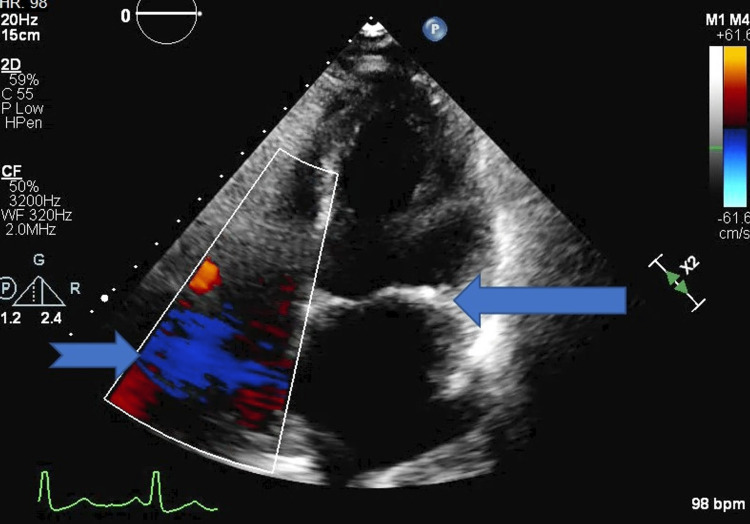
2D echo four-chamber view showing tricuspid and pulmonary regurgitation (blue arrow with broken tail), thickening of mitral valve due to valvulitis (solid blue arrow)

She was admitted, and infectious disease, nephrology, and hematology/oncology consultations were done. She received one unit of packed red blood cell transfusion during her stay. Colonoscopy showed internal hemorrhoids, diverticula, and a 15 mm polyp in the descending colon. She had no active bleeding; upper gastrointestinal endoscopy revealed a small hiatal hernia, a non-obstructing Schatzki ring, and no active bleeding. Anemia workup showed ferritin of 738.5 ng/ml, iron 22 μmol/L, a total iron binding capacity of 118 μmol/L, folate >20 nmol/L, and vitamin B12 of 1,702 pg/ml, and her anemia was thought to be due to chronic disease. All the cultures were negative for any infection. Her renal function remained unchanged (creatinine 2.19 mg/dl [5-25 mg/dL] on admission to 2.31 mg/dl on discharge) despite fluid hydration during her five-day admission. The urine protein-creatinine ratio was 933.1 mg/g; immunofixation was negative for monoclonal free light chains; ANA (anti-nuclear antibodies) 1:720; ANCA (anti-neutrophil cytoplasmic antigens) IgG 1:320; MPO Ab (myeloperoxidase antibody) IgG 179 AU/mL; serine proteinase 30 AU/mL; anti-GBM (anti-glomerular basement membrane) negative; anti-dsDNA (anti-double stranded deoxyribonucleic acid) 1:40; C3 and C4 within normal limits. Based on these, she was diagnosed with granulomatosis with polyangiitis. Echocardiography revealed the left ventricular systolic function is normal with an EF (ejection fraction) of >55%, abnormal left ventricular diastolic function with moderate to severe aortic and mitral regurgitation, mild pulmonic and tricuspid valve regurgitation (Figures 1-4)

**Figure 5:**
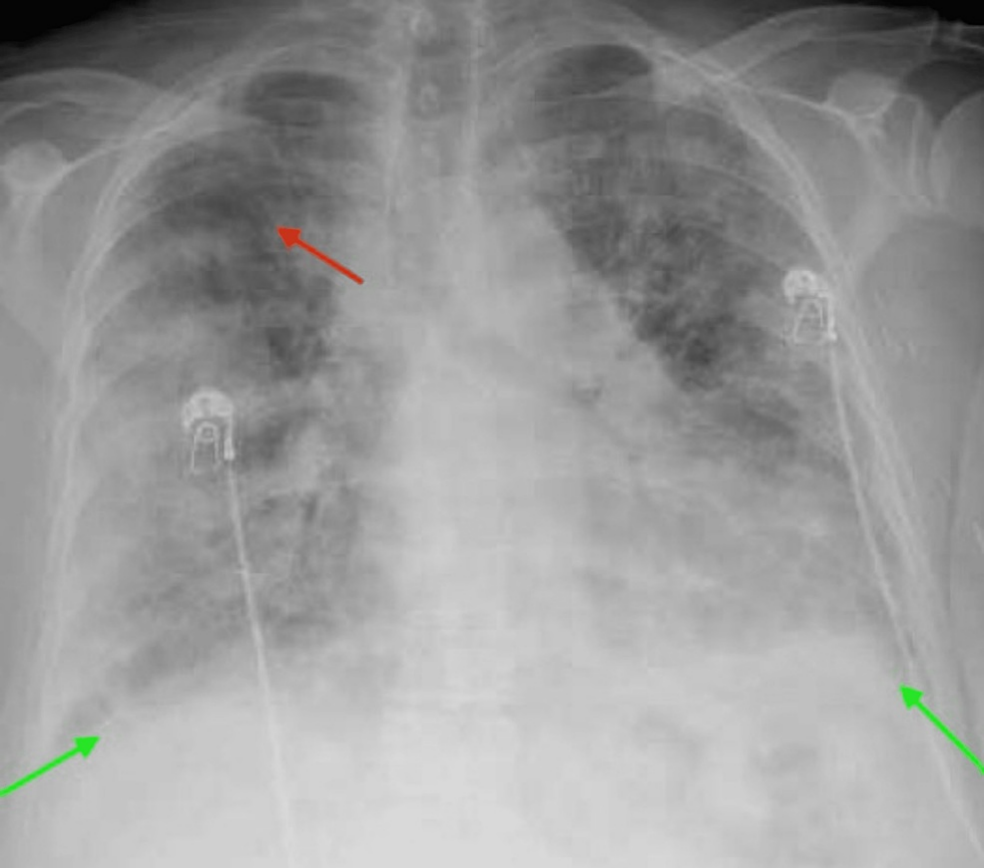
Chest X-ray showing ground-glass opacity diffuse patchy airspace disease compatible with congestion, infection, or ARDS. Red arrow shows cephalization of pulmonary vasculature, green arrow shows blunder costophrenic angles bilaterally. ARDS: acute respiratory distress syndrome

During her stay, she developed a nonproductive cough with shortness of breath and peripheral edema. Repeat laboratory investigations were significant for leucocytosis 30.2 10*3/uL (4.5 - 11.0 10*3/uL), her metabolic profile was significant for blood urea nitrogen of 71 mg/dL ( 5-25 mg/dL), creatinine of 2.56mg/dl (0.44 - 1.00 mg/dL) and EGFR (effective glomerular filtration rate)** **18 ml/min/1.73m*2 ( >60 mL/min/1.73m*2), troponin 0.06 ng/mL (<0.04 ng/mL), BNP (brain natriuretic peptide)** **694 pg/ml (<=100 pg/mL,) C-reactive protein 4.46 mg/dL (0.00 - 0.74 mg/dL), LDH (lactate dehydrogenase)** **381 U/L (91 - 200 U/L), ferritin >1500 ng/ml (11 - 307 ng/mL) and lactic acid of 2.1 mmol/L. Her urinalysis showed mild proteinuria of 30 mg/dL with hematuria. Her chest X-ray showed diffuse patchy airspace disease (Figure 5).

**Figure 6:**
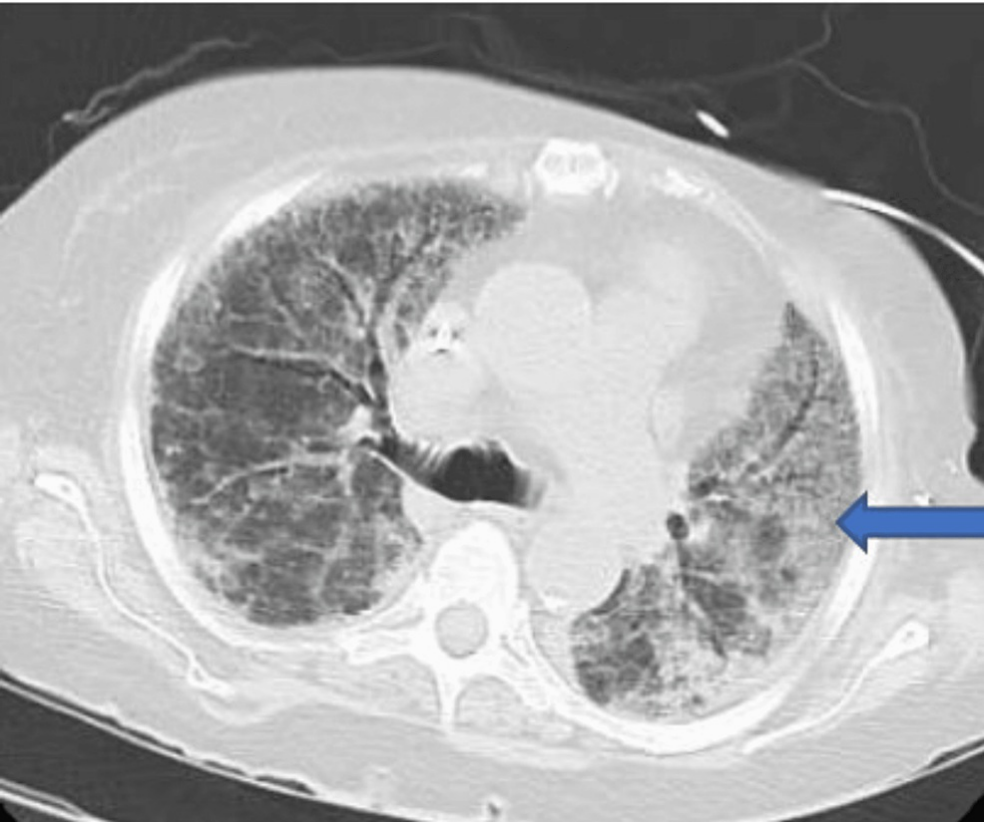
CT chest: blue arrow showing extensive ground-glass infiltrates, extensive fibrotic changes, consolidations at the lung bases, left greater than right.

Computed tomography of the chest without contrast showed extensive ground-glass infiltrates and fibrotic changes. Consolidations at the lung bases were more significant on the left than right (Figure 6).

**Figure 7:**
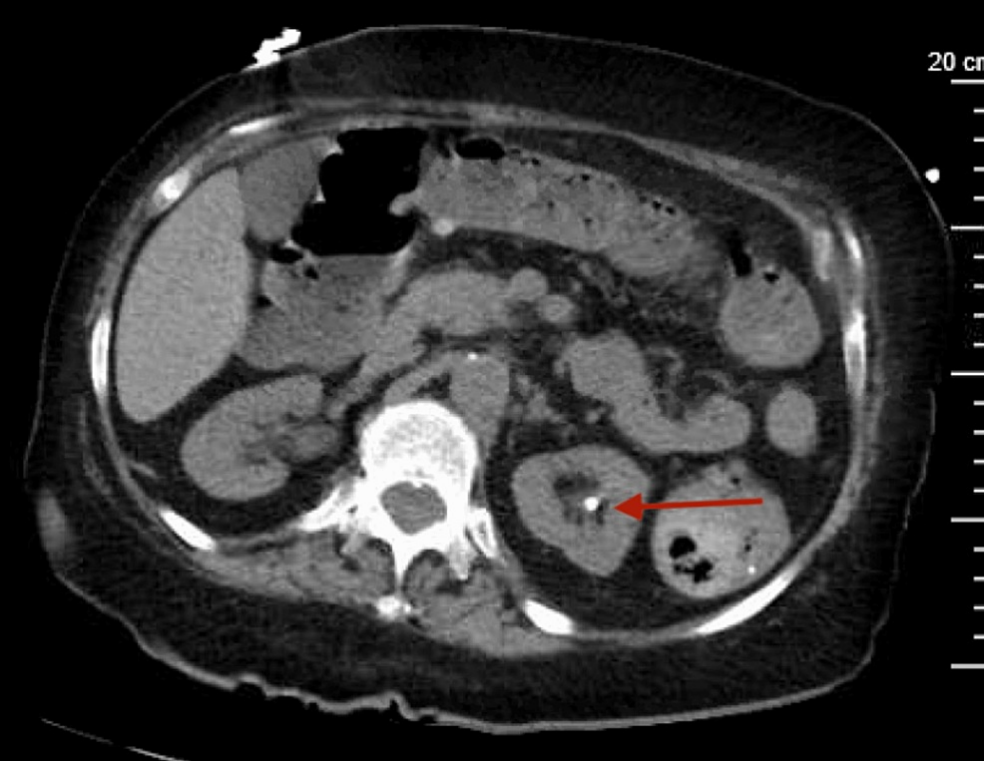
CT abdomen showing 4.7-mm non-obstructing calcification in the left kidney (red arrow)

 It also showed 4.7 mm non-obstructing calcification in the left kidney (Figure 7)

She was started on injection ceftriaxone and azithromycin for pneumonia. She also required oxygen inhalation at 6L through a nasal cannula in view of low oxygen saturation. The renal biopsy showed necrotizing arteritis, multifocal and focal necrotizing, and crescentic glomerulonephritis, a possible immune type (ANCA-associated/clinical). ​​Acute tubular injury, patchy mild tubular atrophy, diffuse interstitial inflammation, and fibrosis were also seen. Based on the renal biopsy, which suggested focal and segmental crescentic glomerulonephritis, she received pulse steroids, plasmapheresis, and rituximab for one month, after which her renal function improved gradually. However, her oxygen saturation continued to be in the 80s, requiring nasal high-flow oxygen. She was later discharged on oxygen to a long-term care facility for further management and weaning with advice to follow up.

## Discussion

The annual incidence of GPA is 10-20 per million worldwide, and the incidence in the United States is reported as 3 per million [4]. GPA is due to proteinase three anti-neutrophil cytoplasmic antibodies (PR3-ANCA), causing inflammation of small vessels. PR3-ANCA is 66% sensitive and 98% specific for GPA and is present in approximately 80%-90% of the patients with active GPA [4]. PR3-ANCA activates neutrophils, inducing de-granulation, damaging endothelial cells, and creating microabscesses [5]. A defective immune response to autoantigens or infection leads to uncontrolled production of Th1 and Th17 cytokines, which can form granulomatous vascular lesions.

GPA commonly involves the upper and lower respiratory tract, systemic vasculitis, kidneys, eyes, and musculoskeletal system and rarely involves the heart. Cardiac involvement in GPA can affect any part, including the coronary artery, conduction pathway, myocardium, endocardium, and pericardium. In 2003, Herbst et al. reported a sessile mass on the anterior leaflet of the mitral valve with regurgitation in a 56-year-old woman, which was initially thought to be an atrial myxoma. However, they discovered an intense inflammatory process that affected the aortic valve and myocardium intra-operatively due to GPA [6]. In 2007, Strizhakov et al. reported a case of a female patient with a GPA who developed third-degree heart block and aortic regurgitation [7]. Cardiac involvement in GPA is associated with a poor prognosis. Hence, these patients should be monitored regularly with systematic and regular cardiac assessments [8]. They can present with the symptoms of shortness of breath, cough, bilateral pedal edema, orthopnea, syncope, and features of heart failure such as elevated jugular venous pressure. Studies have shown that increased cardiac involvement in GPA is associated with a younger age at onset, higher eosinophil count, higher disease activity, and a poorer prognosis [9].

Diagnosis of cardiac involvement in GPA can be made by maintaining a high index of suspicion and following all cases with ECG, TEE (transesophageal echocardiography)​​​​​​, and a thorough history and physical examination. Cardiac MRI has recently become widely recommended by many studies, along with electrocardiography (ECG), echocardiography, and histopathology of the other pulmonary vessels or kidney lesions. ECG and echocardiography can reveal vague features of heart block, valvular stenotic lesions, mass lesions, or regurgitant lesions. Cardiac involvement in GPA is mainly found at autopsy, where destructive changes in the intima and vasculitis with granulomatosis in both the base of the affected valve and severe fibrinous valvulitis of the supravalvular aorta are consistent with GPA. GPA-associated valvular lesions usually present like endocarditis and may pose a diagnostic challenge. Usually, only one valve is affected, and there have been very few reports of multiple valve involvement, as we see in this patient. These valvular lesions cause insufficiency of the affected valve. The aortic valve is most frequently involved, followed by very few cases of mitral involvement. Despite an extensive literature search, we could only find one documented case of the tricuspid valvular lesion with regurgitation. Valvular involvement in GPA usually requires valve replacement [10]. Despite extensive literature searches using PubMed and Google scholar and using keywords, GPA, valvular, cardiac, pulmonary, and tricuspid lesion, we could not find a case of GPA with involvement of all the four valves like in this patient, even though the cardiac involvement is not rare and is present in 6-44% of the cases in recent studies [11]. This patient posed some diagnostic challenges given its atypical presentation and the extent of valvular involvement, which has not been reported before. She had a prolonged hospital stay given worsening cardiac functions and pneumonia with oxygen dependence. This case reinforces the importance of a thorough cardiac evaluation in patients with GPA. Although the treatment of GPA is with cyclophosphamide and corticosteroids, the treatment of cardiac lesions in GPA depends on the type of involvement and varies from case to case. Patients who present with third-degree heart block might be treated with an implantable pacemaker, whereas those with severe fibrinous stenotic or regurgitant lesions are candidates for valvular replacement surgery.

## Conclusions

The cardiac events in granulomatosis polyangiitis are underestimated. Cardiac involvement in GPA can be in many ways, such as heart block, myocarditis, and valvular lesions which are seen mostly in the aortic and mitral valve intima with severe fibrinous vasculitis with granulomatosis in the base of the affected valves. The involvement of the supra-annular aorta is virtually pathognomonic of GPA with cardiac involvement. Even though rare, we found the involvement of tricuspid and pulmonary valves in this patient. This case reinforces the importance of a thorough cardiac evaluation in patients of GPA. The damaged valve will eventually require valve replacement. Hence, it is important for all physicians to regularly monitor and assess GPA patients for any new cardiac symptoms and to treat them early to prevent morbidity and mortality associated with it.
